# Adjusting Lower Plates

**Published:** 1856-10

**Authors:** M. B. Patterson

**Affiliations:** *Dentist*. Saint Paul, Minnesota Territory.


### ARTICLE VIII.

### *Adjusting Lower Plates.*

Messrs. Editors:—In the October number, 1855, of your *valuable* journal, I find a communication from "A Subscriber," with remarks from Dr. White, respecting the adjustment of lower plates; also one from Dr. Goddard in the January number, 1856. Dr. G. very justly remarks that "every member of our profession has his own peculiar method of adjusting plates." As for myself I can claim no very great amount of *unassisted*, practical experience in the adjustment of lower plates; having been in practice away from my preceptor, (Dr. E. P. Byram, of Cooperstown, N. Y.,) only about a year. During the six years I was with him, however, I claim to have seen and assisted in the adjustment of as many complete sets of teeth as usually falls to the lot of a student in dentistry. In that time we (*I* must say *we*, for I had a hand in them all,) adjusted over one hundred lower plates, of which all but one had its mate, the upper plate; and that one never gave satisfaction until it was entirely made anew for the third time. Out of the whole number, we put springs on but one; that was for an old gentleman of sixty years.

We had, perhaps, six of the lower plates to make over, which Dr. Byram attributed to the plates being inserted before absorption of the gums was complete. I will give his manner of adjusting lower plates.

One of the most essential things—and the failure in which is the "rock on which many split"—is to get a *perfect* impression; that is oftentimes a most difficult thing to do, as the wax will in some cases suck itself entirely out of shape in coming off.

To prevent this, in the holders are pierced three small holes of a size to permit the passage of a common broach; one is in the centre of the holder, the others on each side and about one inch from the centre one. Before taking the impression from the jaw, pass a broach through each passage until it touches the ridge of the jaw; that counteracts in a greater or less degree the atmospheric pressure, and as a general thing, the impression, with care, can be taken off undisturbed. Get up the male cast from zinc, the counter-cast from lead, and swage the plate as thick as is possible. I generally use three or four female casts before I get the plate properly swaged. I always want to see an under plate fit as nicely to the jaw as an upper one. If by pressing on one side of a lower plate, the opposite would rise, I do (and when with Dr. Byram *had to do*) the work all over until it was a perfect fit. Every dentist knows that in most cases the lower plate has to be very narrow. Dr. Byram used to tell me, "in making an under plate to cut it until I thought I had spoiled it, and then file off a little more." After trying the plate and being satisfied that the fit is perfect, and that the ordinary movements of the tongue and loose integuments of the mouth will not displace it, proceed to solder around the anterior edge, the half-round wire, and the job is ready for mounting the teeth, which is not the least part of the operation by any means. I consider perfect articulation one of the most essential points in the well fitting of an under plate, especially in mastication.

Another cause of so much trouble from lower plates arises from the great haste which many persons show to "have their teeth" before absorption is half complete, and in some cases actually forcing their dentist to insert the lower plate when he knows that in so doing he is inflicting an injury upon them, and a still greater one upon himself. Much can be said upon adjusting lower plates, and I hope we shall be favored with as many articles on the subject as we have upon plate springing, alias "mysterious twistification."

Before I close I must relate to you a demonstration of genius that does not occur every day. While I was with Dr. Byram, we made a complete set of teeth for a hale, stout, old farmer, by the name of W. After he received the teeth he started for home fearful that he should never learn to use them. Some six weeks afterwards, a neighbor of Mr. W. was in the office, and told us that Mr. W. eat chickens and chicken's bones with his teeth; and furthermore, he had made himself an extra set of teeth for *general use.* The next day Mr. W. himself made his appearance, and sure enough he had made himself an extra set, and had them in his mouth. He had cast them in sand from block tin, using the ones we made him for *moulds.* Both plates were perfect in their fit, and their articulation was beautiful. I often tried to get the Dr. to tell you of it, but he being of a *retiring* and modest disposition, declined.

Respectfully, yours,

M. B. Patterson, *Dentist.*

Saint Paul, Minnesota Territory. *Ib.*

